# Supplementary material for: Active Commuting and Healthy Behavior among Adolescents in Neighborhoods with Varying Socioeconomic Status: The NESLA Study
Source: Int J Environ Res Public Health. 2022 Mar 22;19(7):3784. doi: 10.3390/ijerph19073784 (PMC8997619; doi:10.3390/ijerph19073784)
Supplement: Supplementary file 1 [file ijerph-19-03784-s001.zip › ijerph-1653642-supplementary.pdf]

Supplementary Table S1. Effects of each variable in the final model presented in Table 3.

	Df	Deviance	Resid. Df	Resid. Dev	Wald's Chi-Square
NULL			311	391.41	
Neighborhood SES	2	17.07	309	374.34	14.19***
Are walking or biking ways well illuminated in neighborhood where you live?	1	7.40	308	366.94	4.64*
How often do you eat fruits in a week?	1	4.59	307	362.35	4.16*
How often do you eat junk foods in a week?	1	10.11	306	352.24	9.99**

***p<0.001, **p<0.01, *p<0.05
